# Blau syndrome with persistent fetal vasculature: a case report

**DOI:** 10.1186/s13256-023-03983-1

**Published:** 2023-06-30

**Authors:** Miao Liu, Yong Zeng, Jie Zhong

**Affiliations:** 1 School of Medicine, University of Electronic Science and Technology of China, Chengdu, China; 2 Department of Ophthalmology, Sichuan Provincial People’s Hospital, University of Electronic Science and Technology of China, Chengdu, China

**Keywords:** Blau syndrome, Persistent fetal vasculature, Case report

## Abstract

**Background:**

Blau Syndrome (BS) is a rare autosomal dominant noncaseous granulomatous disease caused by mutations in the NOD2 gene. The disease is characterized by granulomatous dermatitis, symmetrical arthritis, and uveitis, which, if left untreated, can progress to blindness. The diagnosis of BS can be challenging because of its rarity and overlap with other rheumatologic disorders. Early detection of ocular involvement is critical to prevent vision loss and improve the prognosis of patients with BS.

**Case presentation:**

In this report, we present a case of a five-year-old Chinese girl diagnosed with BS one year ago after presenting with a systemic rash and urinary calculi. Genetic testing was recommended by a physician, and a heterozygous mutation of the NOD2 gene c.1538T > C (p.M513T) was identified. Eight months ago, due to bilateral corneal punctate opacity, we had examined and diagnosed bilateral uveitis, bilateral corneal zonal degeneration, persistent fetal vasculature (PFV) in the right eye, and perivascular granuloma in the right eye. As a result, Vitrectomy was performed on the right eye, resulting in a significant improvement in visual acuity from 1/50 on the first day after surgery to 3/10 after 1 week. After 6 months, the visual acuity of the right eye was maintained at 3/20, but opacification of the lens posterior capsule was observed. Follow-up appointments are ongoing to monitor the condition of the affected eyes. Our report underscores the importance of prompt detection and management of ocular involvement in BS accompany with PFV to prevent vision loss and improve patient outcomes.

**Conclusions:**

This report details the case of a child diagnosed with BS who accompanied a periretinal granuloma and PFV in the right eye. Regrettably, the left eye was observed to have no light perception (NLP) with the fundus not being visible. The occurrence of ocular complications in patients with BS, must be closely monitored to prevent vision loss and enhance treatment outcomes. This case underscores the importance of prompt diagnosis and management of ocular complications in patients with BS to prevent further damage and optimize patient outcomes.

## Introduction

Blau Syndrome (BS) is an infrequent autosomal dominant disorder resulting from mutations in the nucleotide-binding oligomerization domain-containing protein 2 (NOD2) gene. Pediatrician Edward Blau first documented cases of BS in 1985 [1]. Currently, seventeen NOD2 gene mutations have been identified as possible causative factors for BS, with the most frequently observed mutations being missense substitutions at position 334 (R334W or R334Q), which affect a highly conserved arginine residue [2]. BS is characterized by the emergence of granulomatous dermatitis, symmetrical arthritis, and recurrent uveitis, which typically manifest before the age of 3–4 years. [1] Atypical cases of BS involving other organs, such as the cardiovascular, nervous, and renal systems, have also been identified [3–5].

The NOD2 gene is situated on chromosome 16 region q12-21 and includes two N-terminal caspase recruitment domains, a nucleotide-binding oligomeric domain, and leucine-rich repeats (LRRs) [6]. The LRRs play a critical role in the identification of muramyl dipeptide (MDP), which activates the nuclear factor-kappaB pathway via oligomerization through the NOD structure. This pathway increases the synthesis of proinflammatory factors such as IL-1b, IL-6, IL-18, and TNF-α, causing persistent macrophage activation and the formation of noncaseous granulomas, leading to autoimmune inflammatory reactions. Understanding the pathophysiology of BS is essential for creating appropriate approaches to help manage this disorder.

## Case presentation

**Fig. 1:**
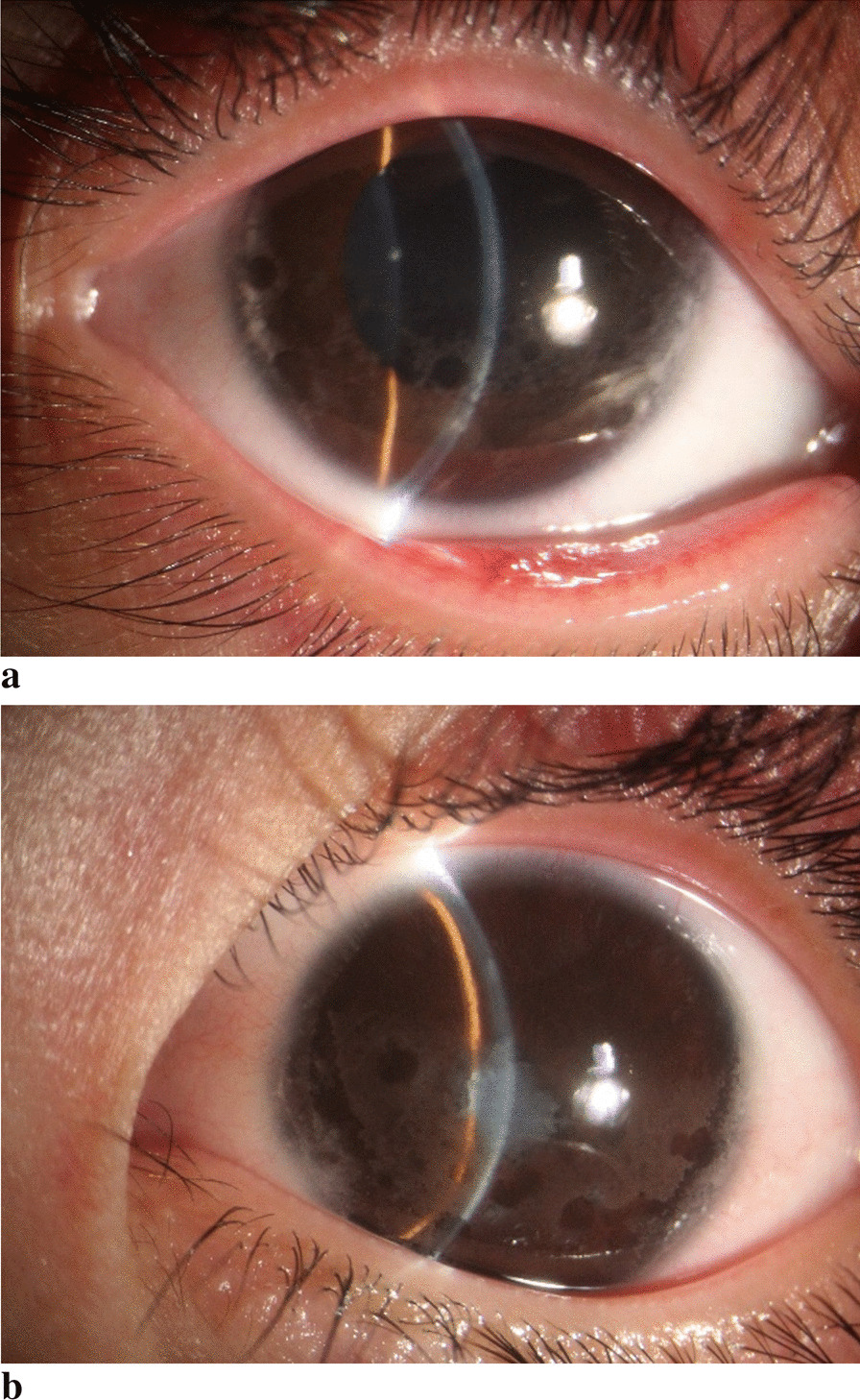
**a** In the photography of the right anterior segment, the cornea of the right eye was patchy, the boundary was clear, and high reflective deposits could be seen behind the cornea. **b** Left anterior segment photography showed lamellar opacity, a clear boundary, KP(+), a shallow anterior chamber, an irregular pupil and lens opacity in the left eye

A five-year-old Chinese girl presented to our hospital with spotty and turbid corneas in both eyes, which had been ongoing for two months, as indicated by her parents. Upon examination, the child’s best corrected visual acuity in the right eye was 20/50, while the left eye was blind. Further investigation revealed that both eyes displayed zonal corneal degeneration, characterized by hyper-reflective deposits behind the cornea. In the right eye, a round pupil with satisfactory light reflection and clear lenses were observed (Fig. 1a). However, in the left eye, the anterior chamber was shallow, with posterior synechia of the iris, and a turbid crystal. The pupil of the left eye was irregular, with no detectable light reflection (Fig. 1b), and the fundus remained unclear.

**Fig. 2:**
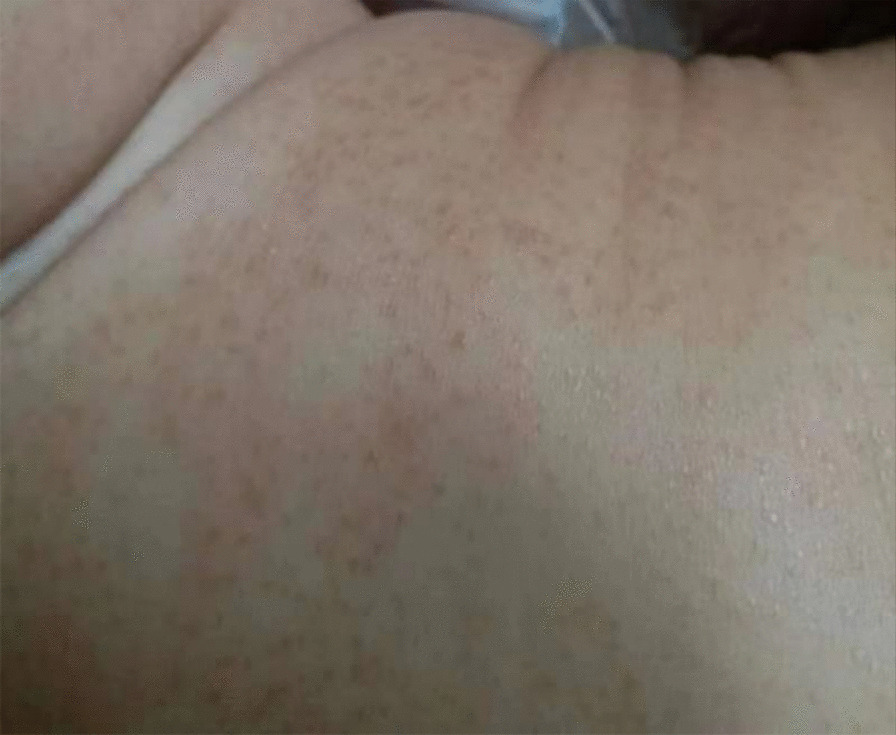
Large nodular papules were seen on the skin of the child

One year prior, the child was hospitalized for malnutrition and nodular papules (Fig. 2). A skin biopsy revealed granuloma-like structures with mononuclear histoid cells and multinucleated giant cells in the dermis, accompanied by red-stained collagen-like material and inflammatory cell infiltration. A color ultrasound examination of the urinary system showed medullary sponge kidney, a congenital dysplasia characterized by spindle or small capsule expansion in the medullary collecting duct and pyramidal papillary duct of one or more papillary kidneys, leading to infection and urinary calculi formation in most cases. Genetic testing revealed a heterozygous mutation (c.1538T > C) in the NOD2 gene leading to the conversion of amino acid 513 from lysine to threonine (p.M513T), while the child's parents had no mutation at that site. The diagnosis of BS was established, and adalimumab was prescribed. However, the child was re-hospitalized several times due to mycoplasma pneumonia and developed a cyst in her knee joint.

**Fig. 3:**
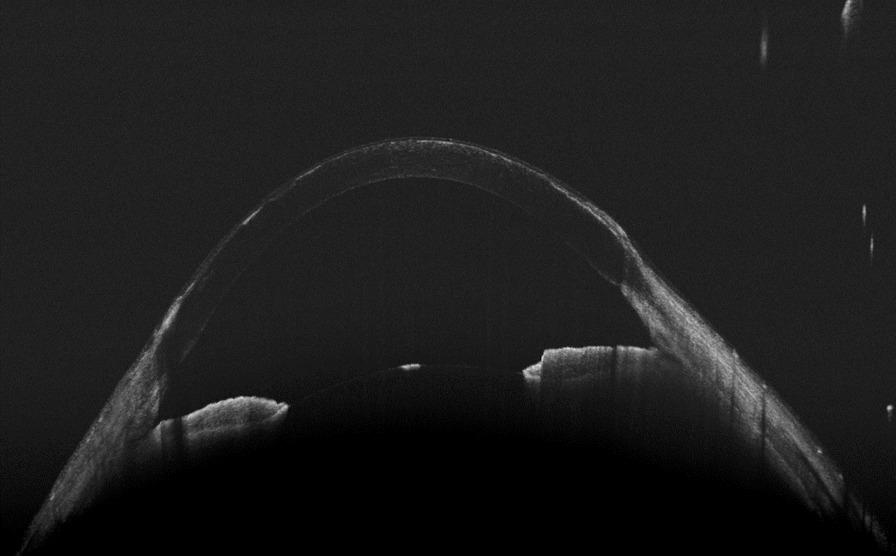
OCT of the anterior segment showed a high reflex in the anterior part of the nasal and temporal cornea, corresponding to the focus of corneal infiltration

**Fig. 4:**
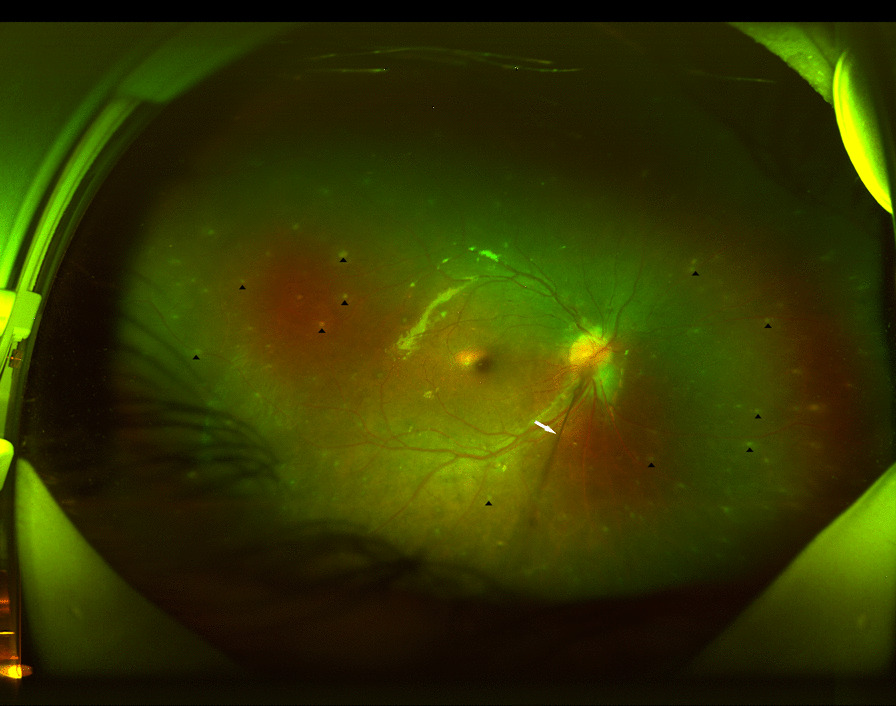
In ultra-wide-field fundus photography, the retina of the right eye contained scattered off white punctate lesions, and a cord-like structure protruded into the vitreous cavity in front of the optic papilla

**Fig. 5:**
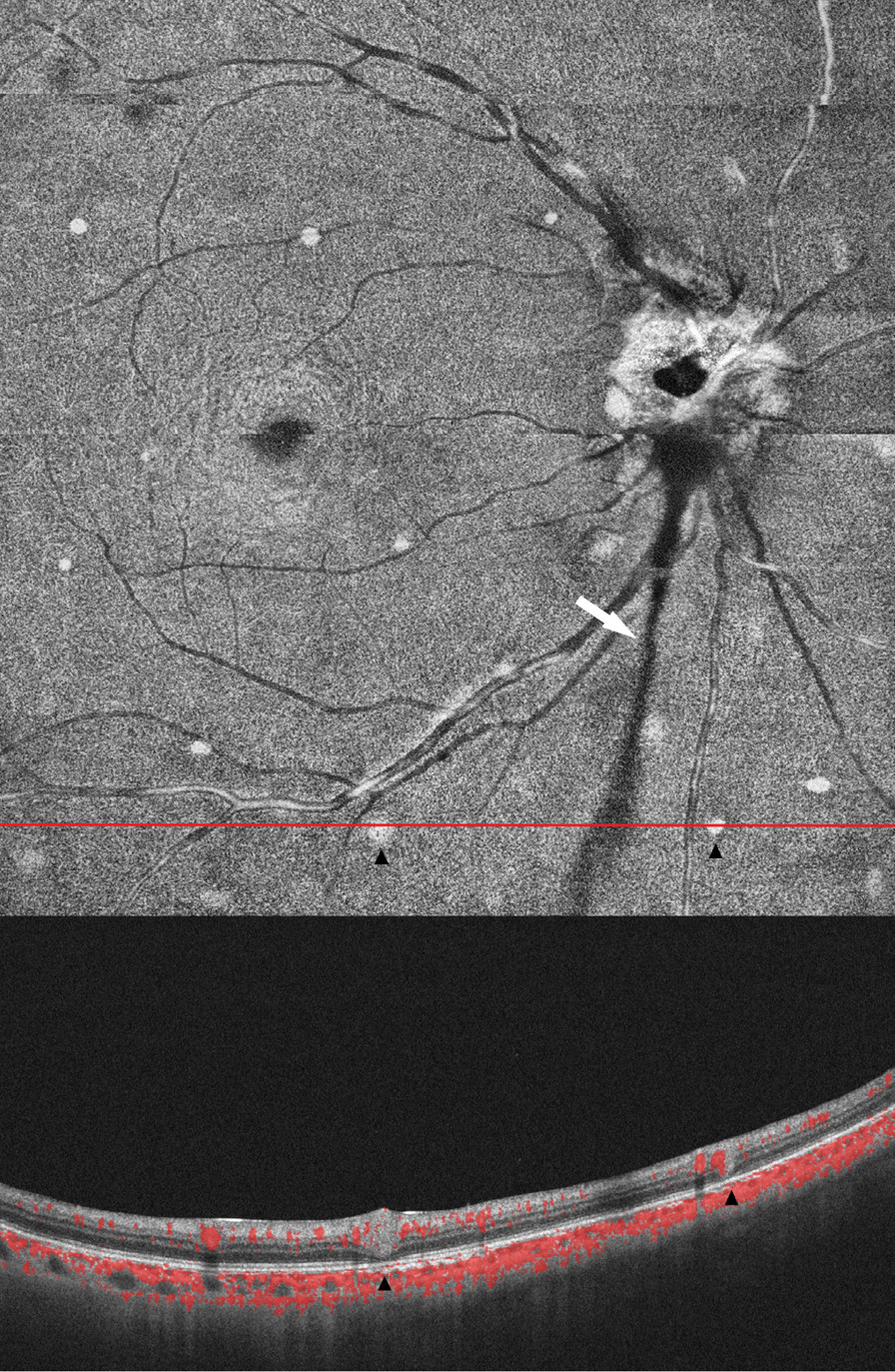
OCTA:EN-FACE scans of the right eye showed a perivascular round nodular lesion corresponding to ultrawide-angle fundus photography. A B-SAN scan showed that the lesion was located in the retinal neuroepithelium, and some blood flow signals could be seen

**Fig. 6:**
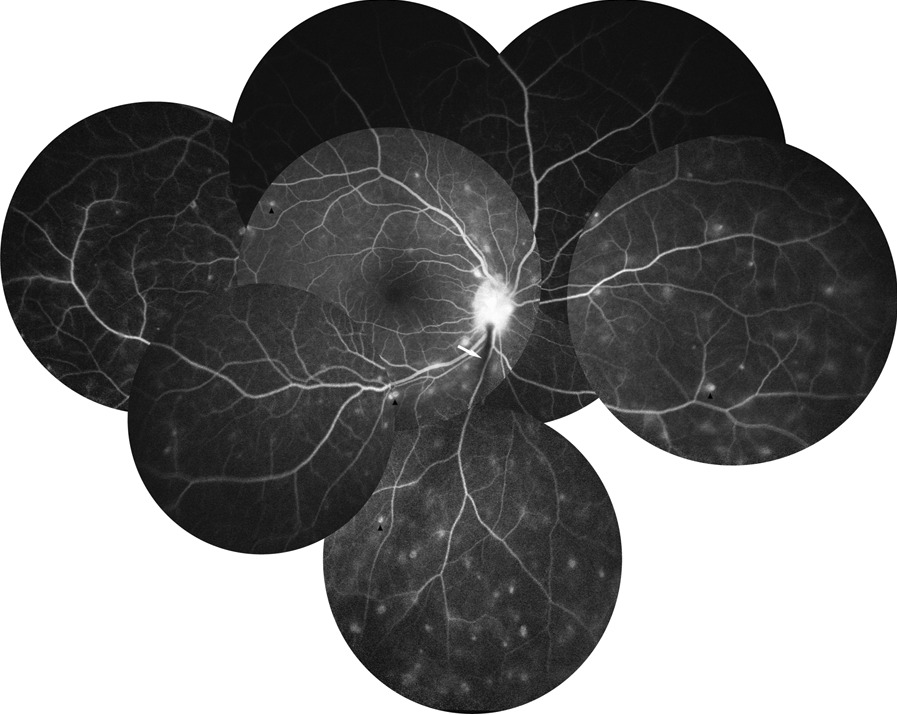
Fluorescein angiography of the right eye showed that the surface of the optic papilla was covered by fluorescence, the optic papilla was strongly fluorescent, and the boundary was unclear. Each quadrant was scattered with high fluorescence, the boundary was slightly blurred, and telangiectasia with microleakage was observed

**Fig. 7.**
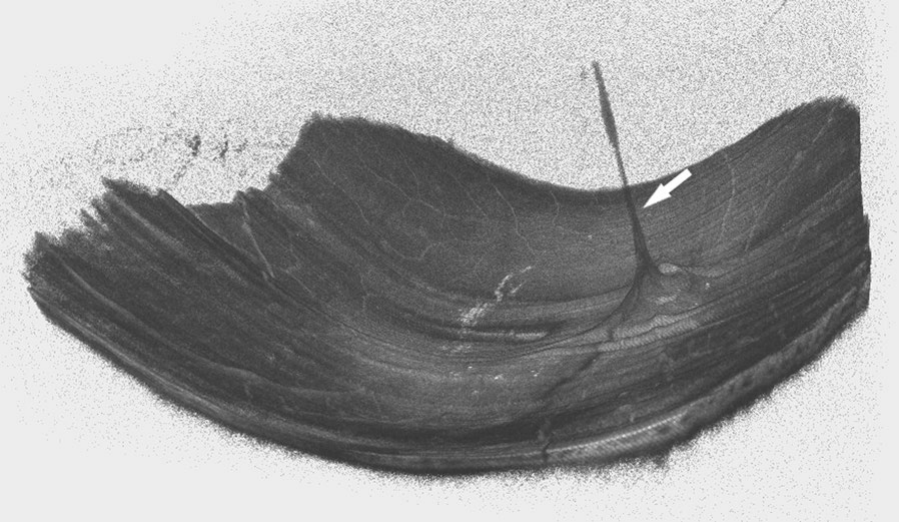
3D image of the retina of the right eye: A cord-like structure of the right eye extended from the optic papilla to the vitreous cavity

Anterior segment OCT examination revealed anterior corneal hyperreflexes in both eyes (Fig. 3), while fundus photography showed scattered off-white punctate lesions throughout the retina (Fig. 4). OCTA EN-FACE scans revealed these round lesions to be situated beside retinal vessels (Fig. 5). B-SCAN imaging exhibited high reflective nodular changes within the retina, while fundus fluorescein angiography showed scattered dim high fluorescent lights in each quadrant of the venous phase, with slightly blurry boundaries (Fig. 6). In addition, the imaging revealed masking of the right optic disc surface fluorescence in strips, along with dilated capillaries with microleakage surrounding the retina. Through 3D and ocular B-ultrasound evaluation of the right eye, a ribbon-like structure extending from the optic papilla to the vitreous cavity was apparent (Fig. 7). Routine blood examinations recorded elevated leukocytes, while urine analysis reported protein (+ +), with no significant changes in liver and kidney functions, coagulation function, or blood transfusion tests.

To prevent lens capsule rupture and retinal detachment caused by PFV, we performed vitrectomy on the right eye, in conjunction with oral hormone therapy to manage the development of BS. Post-operatively, corneal edema was observed, with a visual acuity of 1/50 in the right eye on the first day, which improved to 3/10 after one week. Following the 6-month follow-up, the child’s left eye was atrophied, with corneal endothelial decompensation, while the right eye’s vision was maintained at 3/20. Furthermore, the right lens posterior capsule showed opacification, and we continued to monitor the condition of the affected eyes. This case report emphasizes the crucial role of diagnostic imaging in identifying the ocular complications affiliated with BS, particularly PFV. Early intervention, such as vitrectomy, combined with oral hormone therapy, can improve patient outcomes. Continual long-term follow-up and proper management of ocular complications are necessary to prevent visual impairment in patients with BS.

## Discussion

BS typically manifests initially as arthritis, [7] which can lead to misdiagnosis with juvenile rheumatoid arthritis (JRA) in the absence of ocular and cutaneous symptoms. Unlike JRA, which causes anterior uveitis in only 10–20% of patients, panuveitis with multifocal choroidal scars is common in patients with BS, occurring in almost 75% of cases [8]. Here, we present a case study of a patient with a history of rash, arthritis and binocular uveitis, whose genetic testing revealed a heterozygous mutation in the NOD2 gene, leading to a BS diagnosis. The fundus examination (Fig. 4) showed no signs of multifocal choroiditis markers, but instead depicted scattered yellowish-white lesions upon ultra-wide fundus photography. OCTA examination of the patient (Fig. 5) revealed that these lesions predominantly surrounded the retinal vessels, exhibited high reflection in the retina, and had some detectable blood flow signals. Gass [9] reported the presence of nodular wax droplet exudate around the retinal vessels in the retinal tissue of sarcoidosis patients as a result of epithelioid cell proliferation. Wong and Erika [10, 11] utilized optical coherence tomography to show the existence of intraretinal perivascular nodules that extend to the anterior retinal space. Considering the granulomatous nature of BS, the high reflex points of the fundus observed in our patient may be associated with periretinal granuloma, a rare finding in granulomatous diseases. This case highlights the complexity of distinguishing BS from other rheumatologic disorders, particularly with atypical ocular manifestations. While multifocal choroiditis is a classical ocular presentation that can aid in the diagnosis of BS, cases like ours underscore the need for advanced imaging technologies, such as OCTA, to differentiate BS from other granulomatous diseases.

Our case presents a rare occurrence of BS in combination with PFV. After reviewing previous case reports, we did not discover any documented cases of BS with PFV. PFV is a congenital disease caused by incomplete degeneration of the primordial vitreous in the embryonic stage. There are fourteen key genes [12] (TP53, VEGFA, Smad2, and so on) associated with it, but our patient’s case did not exhibit any genes related to PFV. The cord-like proliferation surrounding the optic papilla, found in this instance, may be associated with granulomatous inflammation of the optic nerve head, according to Ester's study [13]. However, the relationship between PFV and BS is still unclear.

Furthermore, due to the rarity of BS and the high cost of genetic testing, BS is frequently misdiagnosed in clinical settings. In addition, the disease is often misdiagnosed as juvenile idiopathic arthritis and sarcoidosis before the appearance of eye manifestations, which leads to a significant reduction in the diagnostic rate of the disease. At present, there is no clear treatment plan for the disease. The effect of low-dose glucocorticoids in the quiescent period is usually good [6]. It has been reported that glucocorticoids are combined with immunosuppressants, but the effect is not exact. Thalidomide is used to inhibit the KF-κB pathway and can control inflammation, [14] which is a promising research direction. Tumor necrosis factor-α antagonists and interleukin-1 inhibitors have also shown good efficacy in treatment [3, 6].

## Conclusion

In summary, we report a case of BS with typical manifestations of arthritis, rash and uveitis, but this patient also had a rare perivascular granuloma of the retina and PFV findings. Her left eye had no light perception, and the eyeball was slightly atrophied. Hence, the ocular complications associated with BS must be taken into account to prevent unfavorable visual outcomes.

## Data Availability

Not applicable.
